# Prognostic significance of SATB1 in gastrointestinal cancer: a meta-analysis and literature review

**DOI:** 10.18632/oncotarget.16867

**Published:** 2017-04-05

**Authors:** Sheng Zhang, Yi Xin Tong, Xiang Shang Xu, Hui Lin, Teng Fei Chao

**Affiliations:** ^1^ Department of Gastrointestinal Surgery, Tongji Hospital, Tongji Medical College, Huazhong University of Science and Technology, Wuhan, China; ^2^ Tongji University, School of Medicine, Shanghai, China; ^3^ Department of Oncology, Tongji Hospital, Tongji Medical College, Huazhong University of Science and Technology, Wuhan, China

**Keywords:** SATB1, gastrointestinal cancer, meta-analysis, overall survival, relapse free survival

## Abstract

**Background:**

The special AT-rich sequence-binding proteins 1 (SATB1) is a major regulator involved in cell differentiation. It has been shown that SATB1 acts as an oncogenic regulator. The clinical and prognostic significance of SATB1 in gastrointestinal cancer remains controversial. The purpose of this study is to conduct a systematic review and meta-analysis to elucidate the impact of SATB1 in gastrointestinal cancer.

**Results:**

A total of 3174 gastrointestinal cancer patients from 15 studies were included. The correlation between SATB1 expression and OS or RFS was investigated in 12 and 5 studies respectively. The results of meta-analysis showed that SATB1 overexpression is inversely correlated with OS (combined HR: 1.79, *p* = 0.0003) and RFS (combined HR: 2.46, *p* < 0.0001). In subgroup analysis, SATB1 expression is significantly correlated with poor prognosis in gastrointestinal cancer in Asian population. SATB1 expression is associated with stage, invasion depth, lymph node metastasis and distant metastasis.

**Methodology:**

Published studies with data on overall survival (OS) and/or relapse free survival (RFS) and SATB1 expression were searched from Cochrane Library, PubMed and Embase (up to Dec 30, 2016). The outcome measurement is hazard ratio (HR) for OS or RFS related with SATB1 expression. Two reviewers independently screened the literatures, extracted the data and performed meta-analysis using RevMan 5.3.0 software. The combined HRs were calculated by fixed- or random-effect models.

**Conclusions:**

The results of this meta-analysis suggest that SATB1 overexpression is related to advanced stage, lymph node metastasis and distant metastasis. SATB1 overexpression is a marker indicating poor prognosis in gastrointestinal cancer.

## INTRODUCTION

Colorectal cancer (CRC), gastric cancer (GC) and esophageal cancer (EC) are three major malignancies in the gastrointestinal tract, of which CRC is the second leading cause of cancer death in the United States and the third most frequent cancer worldwide. Gastric cancer and esophageal cancer are relatively rare but have poorer prognosis, with only 30.4% and 18.4% five year survival rate in United States respectively [1–2]. Cancer is a heterogeneous disease involving multiple genetic and epigenetic factors, thus provides many challenges in anti-cancer treatment. For instant, in colorectal cancer, although several novel target therapies (i.e., bevacizumab and cetuximab) have been developed and proven to be effective, there is still a large portion of patients who respond poorly to these target therapies. Therefore, it is crucial to identify new prognostic markers and potential therapeutic targets for those who failed to respond to the existing treatments [3].

SATB1 (The special AT-rich sequence-binding proteins 1) is a nuclear matrix associated proteins which is a transcription factor involved in chromatin remodeling and gene regulation [4]. In physiological conditions, SATB1 plays a pivotal role in cell differentiation and thymocyte development [5, 6]. Recent studies have also shown that SATB1 is involved in tumor development, progression and metastasis. Clinical studies showed that SATB1 is overexpressed in various types of cancer such as breast cancer [7], endometrial cancer [8] and renal cell carcinoma [9], etc. Overexpression of SATB1 correlated with adverse clinical parameters and poor prognosis. *In vitro* study demonstrated that SATB1 can promote pancreatic cancer cell growth and invasion through the induction of oncogene MYC mRNA and protein expression [10]. Tesone AJ et al. reported that the overexpression of SATB1 could initiate tumor-promoting activities in cancer-associated dendritic cells, which may contribute to the progression of malignancy [11]. In prostate and bladder cancer, overexpression of SATB1 can induce epithelial-mesenchymal transition (EMT) which leads to cancer cell invasion and metastasis [12, 13].

In colorectal cancer, Mir R et al [14] demonstrated that SATB1 is a novel target of Wnt/β-catenin signaling. They showed that in colorectal cells, SATB1 regulates multiple downstream effectors and mediators of Wnt pathway which promote an aggressive phenotype and tumorigenesis. Moreover, Frömberg and colleagues reported that knockdown of SATB1 in colorectal cell lines can interfere with numerous gene expressions such as *MMP7, VEGF*, N-cadherin, Slug, Twist, β-catenin etc, which involve in proliferation, cell cycle, EMT, invasion and cell survival [15].

Althought it is still controversial whether SATB1 could play a complex molecular role of tumor-promoting and possible inhibitory effects in carcinogenesis by affecting multiple pathways [15], it is necessary to obtain high level evidence-base results to determine the prognostic value of SATB1 in gastrointestinal malignancy patients and identify subgroup of patients that could potentially benefit from target therapy. In this study, we performed a systematic review of literatures and meta-analysis to determine the association between SATB1 expression and overall survival (OS) in colorectal, gastric and esophageal cancer. We also analyzed SATB1 expression and its relation to the clinicopathological characteristics such as TNM stage, lymph node involement and tumor differentiation.

## RESULTS

### Search results and description of studies

**Figure 1:**
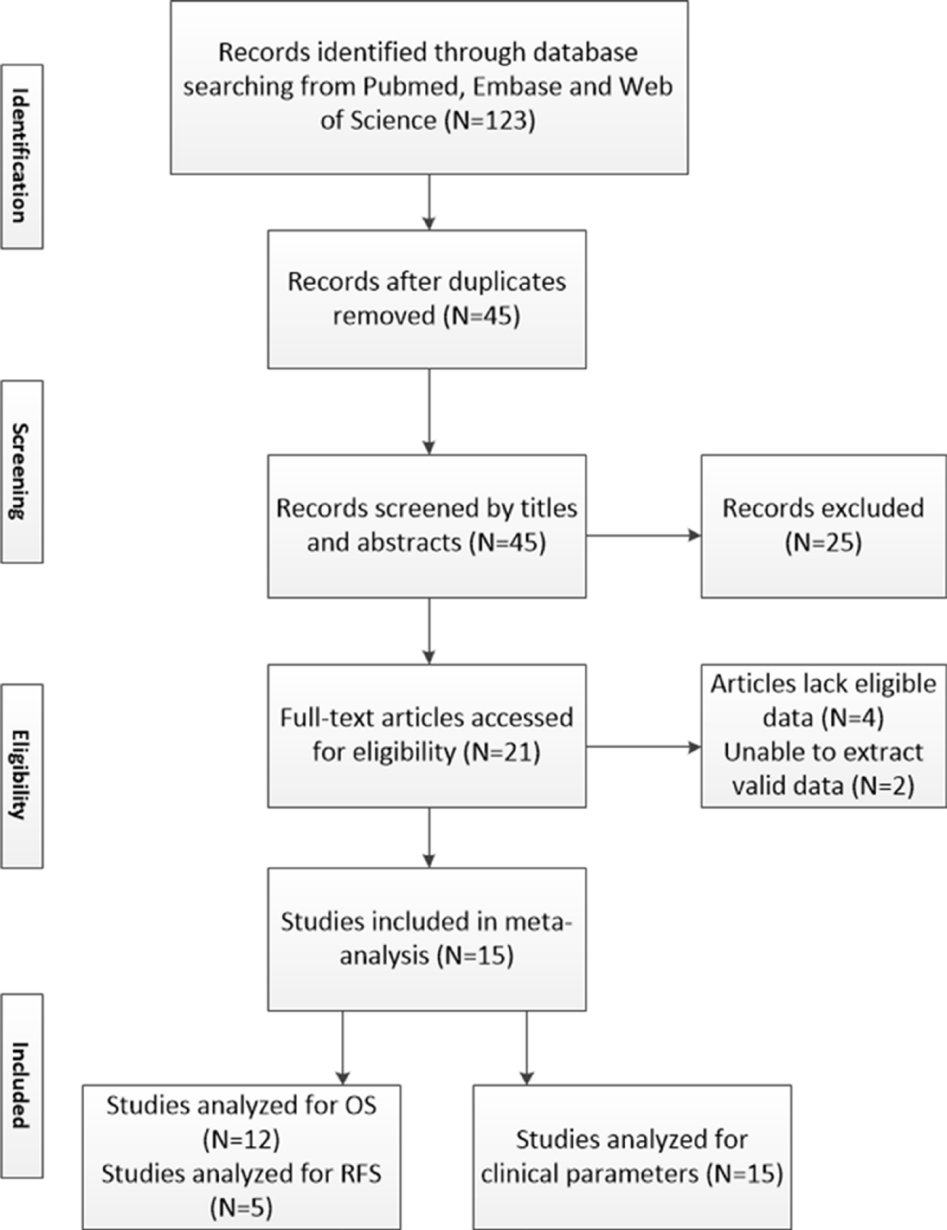
Brief flow chart N = numbers of study; OS = overall survival; RFS = relapse free survival.

In total, 123 studies have been identified by our search, of which 56 articles were from Pubmed and 67 were from other databases (Figure 1). After removal of duplication and screening of titles and abstracts, 20 articles were potential eligible for our meta-analysis. After careful reading, 15 studies fulfilled our inclusion criterias [16–30]. Among the included articles, 12 studies [16–21, 25–30] had available data to analyze OS and 5 studies [16, 21, 22, 25, 30] had available data to analyze RFS. In terms of cancer type, 9 studies [16–24] contained or included data of SATB1 expression in CRC patients, 4 studies [25–28] with data on SATB1 expression in GC patients, 2 studies with SATB1 expression in other types of gastrointestinal cancer, more specifically in esophageal cancer [29] and pancreatic cancer [30]. All included studies are summarized in Table 1.

**Table 1:** Characteristics of studies included in meta-analysis

First Author	Year	Country	Cancer Type	Patient Number	Stage	Median Follow-up (months)	Method	Cut-off	HR estimation	Statistic	HR (95% CI) of OS	Quality Score (%)
Sun [16]	2015	China	Rectal	132	I–IV	75	IHC	IRS ≥ 1	given by author	Multi-variate	6.25 (1.68–23.28)	32 (80%)
Kowalczyk [17]	2015	Poland	CRC	102	I–IV	36.2	IHC	IRS > 1	given by author	Multi-variate	1.69 (0.89–3.55)	31 (78%)
Zhang [18]	2014	China	CRC	520	I–IV	N/A	TMA	Intensity of nucleus	given by author	Multi-variate	1.45 (1.09–1.91)	29 (73%)
Al-Sohaily [19]	2013	Australia	CRC	352	I–IV	66	TMA	Mean nuclear staining > = 5%	given by author	Multi-variate	0.63 (0.43–0.92)	32 (80%)
Niu [20]	2014	China	CRC	131	I–IV	56	IHC	IRS ≥ 1	given by author	Multi-variate	1.77 (1.03–3.03)	35 (88%)
Nodin [21]	2012	Sweden	CRC	626	I–IV	40.2	TMA	IRS ≥ 1	given by author	Multi-variate	2.05 (1.09–3.88)	32 (80%)
Hironobu [22]	2016	Japan	CRC	328	I–III	62	IHC	IRS ≥ 1	given by author	Multi-variate	2.34 (1.5–3.65)	33 (83%)
Zhang [23]	2013	China	CRC	80	I–IV	N/A	IHC	≥ 25% positive nuclei	N/A	N/A	N/A	N/A
Meng [24]	2011	China	Rectal	93	I–IV	N/A	IHC	IRS ≥ 1	N/A	N/A	N/A	N/A
Hedner [25]	2014	Sweden	GC & EC	175	I–IV	62.4	TMA	IRS ≥ 1	given by author	Multi-variate	2.3 (1.32–4.01)	33 (83%)
Lu [26]	2010	China	GC	118	I–IV	N/A	IHC	propotion ≥ 25%	given by author	Multi-variate	1.71 (1.08–2.72)	33 (83%)
Yuan [27]	2016	China	GC	60	I–IV	N/A	QPCR	2−ΔΔCT	Calculated from Median survival	Uni-variate	1.87 (0.90–3.87)	24 (60%)
Chen [28]	2010	China	GC	102	I–IV	34	IHC	Intenstity + proportion > 2	given by author	Multi-variate	1.79 (1.08–2.96)	32 (80%)
Cong [29]	2015	China	EC	180	I–III	N/A	IHC	IRS > 4.5	Calculated from K-M curve	Multi-variate	3.56 (2.19–5.79)	30 (75%)
Elebro [30]	2014	Sweden	PC	175	I–IV	N/A	TMA	IRS ≥ 1	given by author	Multi-variate	1.79 (1.05–3.05)	31 (78%)

IRS = SI (staining intensity) ×PP (percentage of positive cells). SI was determined as 0, negative; 1, weak; 2, moderate; and 3, strong. PP was defined as 1, 0–9% positive cells; 2, 10–50% positive cells; and 3, > 50% positive cells. One hundred cells were counted in each of ten high-power visual fields (40×) from different areas of each section chosen at random for IRS evaluation, and the average IRS was calculated. The final intensity of SATB1 staining was defined as ‘negative’ and ‘positive’, corresponding to IRS values of ≤ 1 and > 1, respectively.

### Impact of SATB1 expression on prognosis in all patients

**Figure 2:**
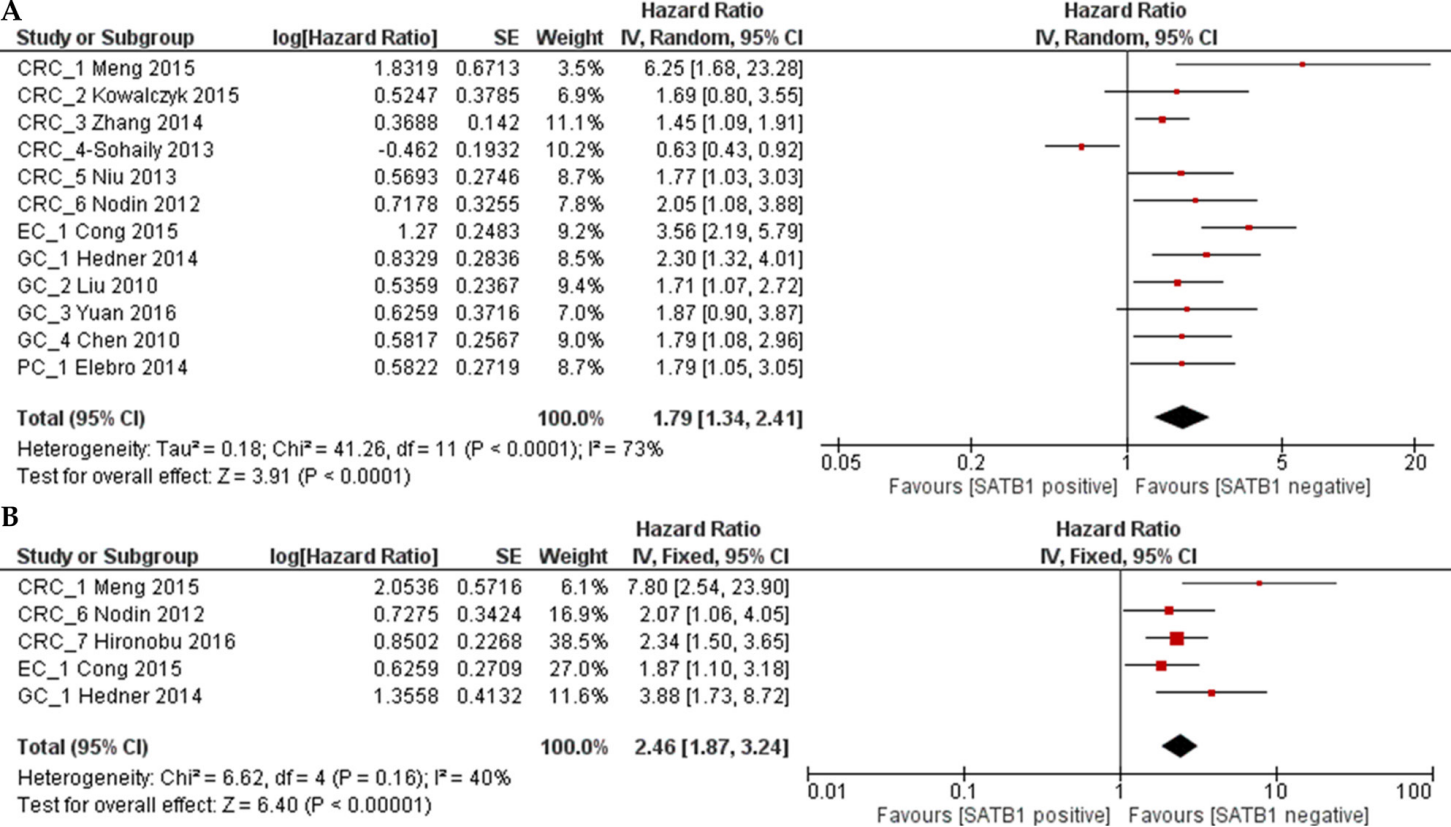
Forest plot of the hazard ratio (HR) for overall survial (OS) (**A**) or relapse free survival (RFS) (**B**) associated with SATB1 expression in all gastrointestinal cancer patients.

**Figure 3:**
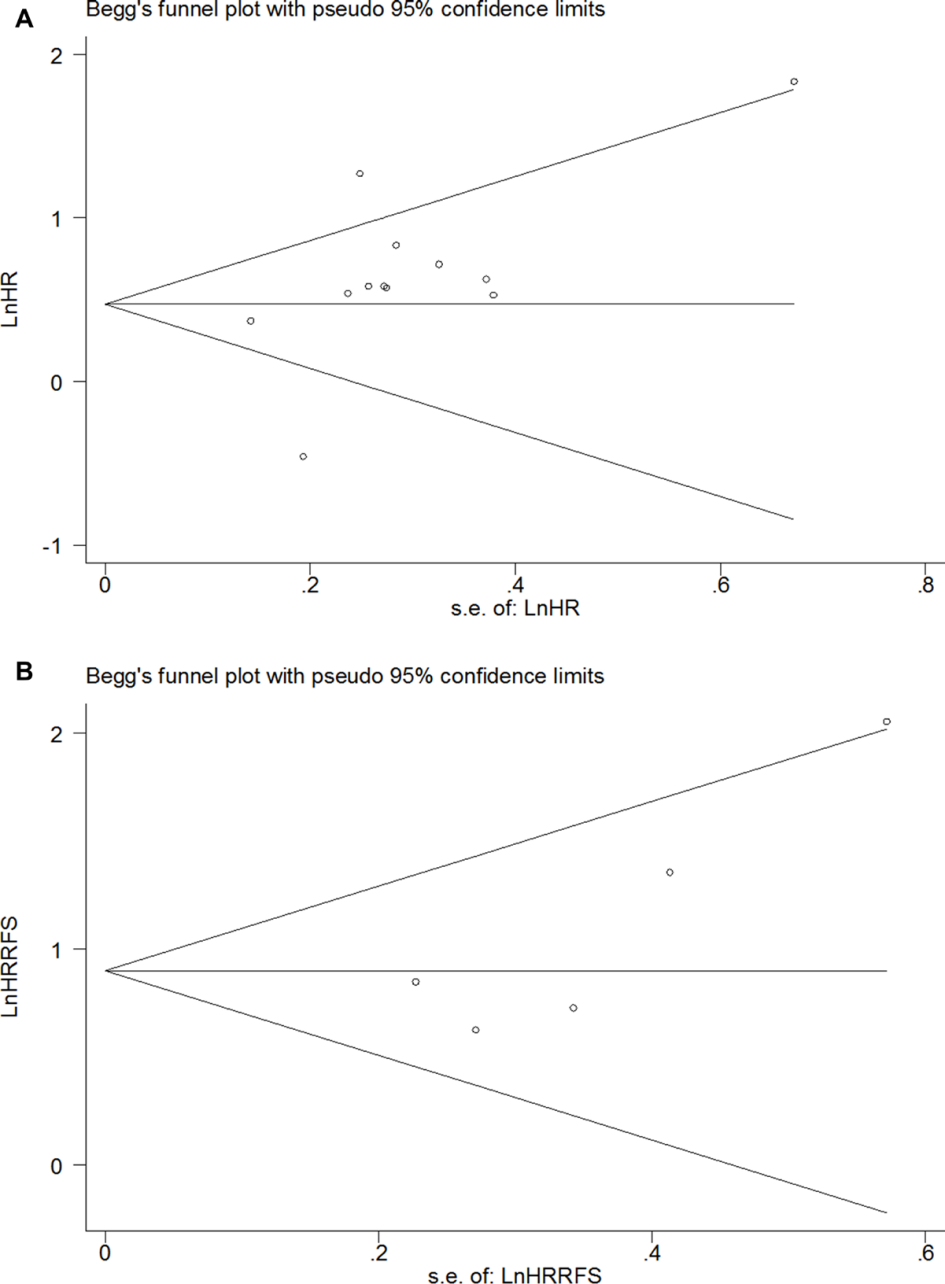
Funnel plot of the HR for OS (**A**) and RFS (**B**) in all gastrointestinal cancer patients.

OS was analyzed in 12 studies with 2673 patients. Random effect model was used as there is significant heterogeneity among studies (*p* < 0.0001). As shown in Figure 2A, the combined HR was 1.79 (95% CI 1.34–2.41, *p* = 0.0003), which indicated that SATB1 overexpression is associated with a 1.79 fold increase in mortality in gastrointestinal tract cancer. Begg's test and Egger's test showed no significant publication bias (*p* = 0.19 and *p* = 0.09) (Figure 3A). RFS was only reported in five studies and the combined HR was 2.46 (95% CI 1.87–3.24, *p* < 0.0001), which indicated that SATB1 overexpression is associated with 2.46 fold increased risk of cancer relapse in gastrointestinal tract cancer (Figure 2B). There was no significant publication bias based on Begg's (*p* = 0.22) and Egger's test (*p* = 0.09) (Figure 3B).

### Impact of SATB1 expression on OS of western and Asian patients

**Figure 4:**
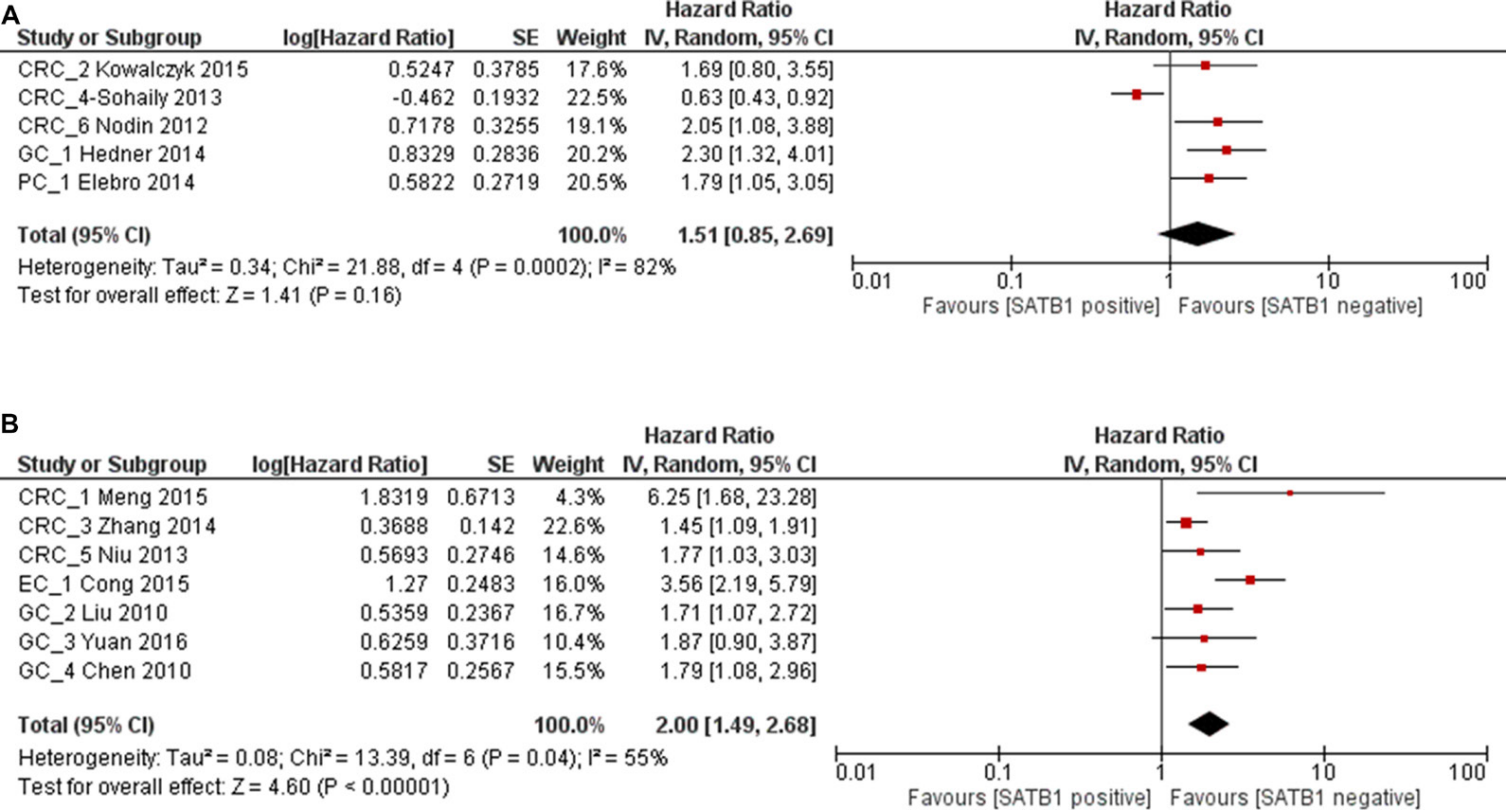
Forest plot of HR for OS associated with SATB1 expression in Western population (**A**) and Asian population (**B**).

**Figure 5:**
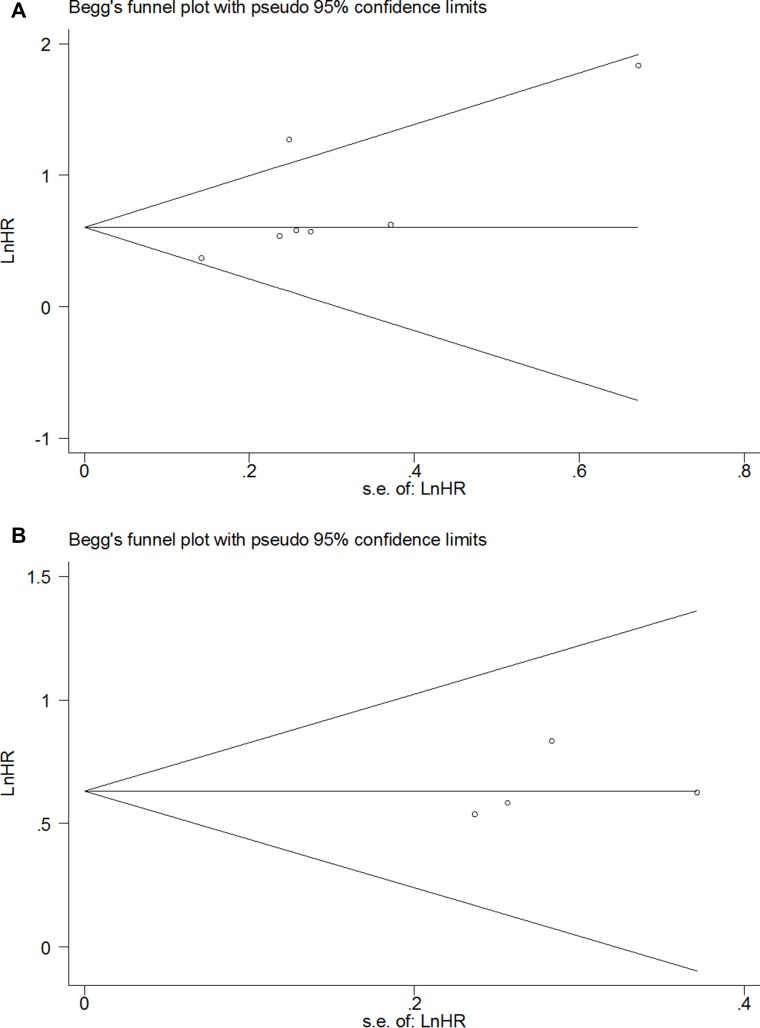
Funnel plot of the HR for OS in Asian patients (**A**) and GC patients (**B**).

In subgroup analysis, the impact of SATB1 on OS in Western patients was evaluated in 5 studies. The combined HR was 1.51 (95% CI 0.85–2.69), however it was not statistically significant (*p* = 0.16) (Figure 4A). In Asian population, the combined HR of SATB1 positive expression on OS was 2.00 (95% CI 1.49–2.68, *p* < 0.00001), which suggested that overexpression of SATB1 predicted poor prognosis in Asian population (Figure 4B). Begg's test and Egger's test showed there was no significant publication bias (*p* = 0.133 and *p* = 0.107) (Figure 5A).

### Impact of SATB1 expression on OS in CRC and GC patients

**Figure 6:**
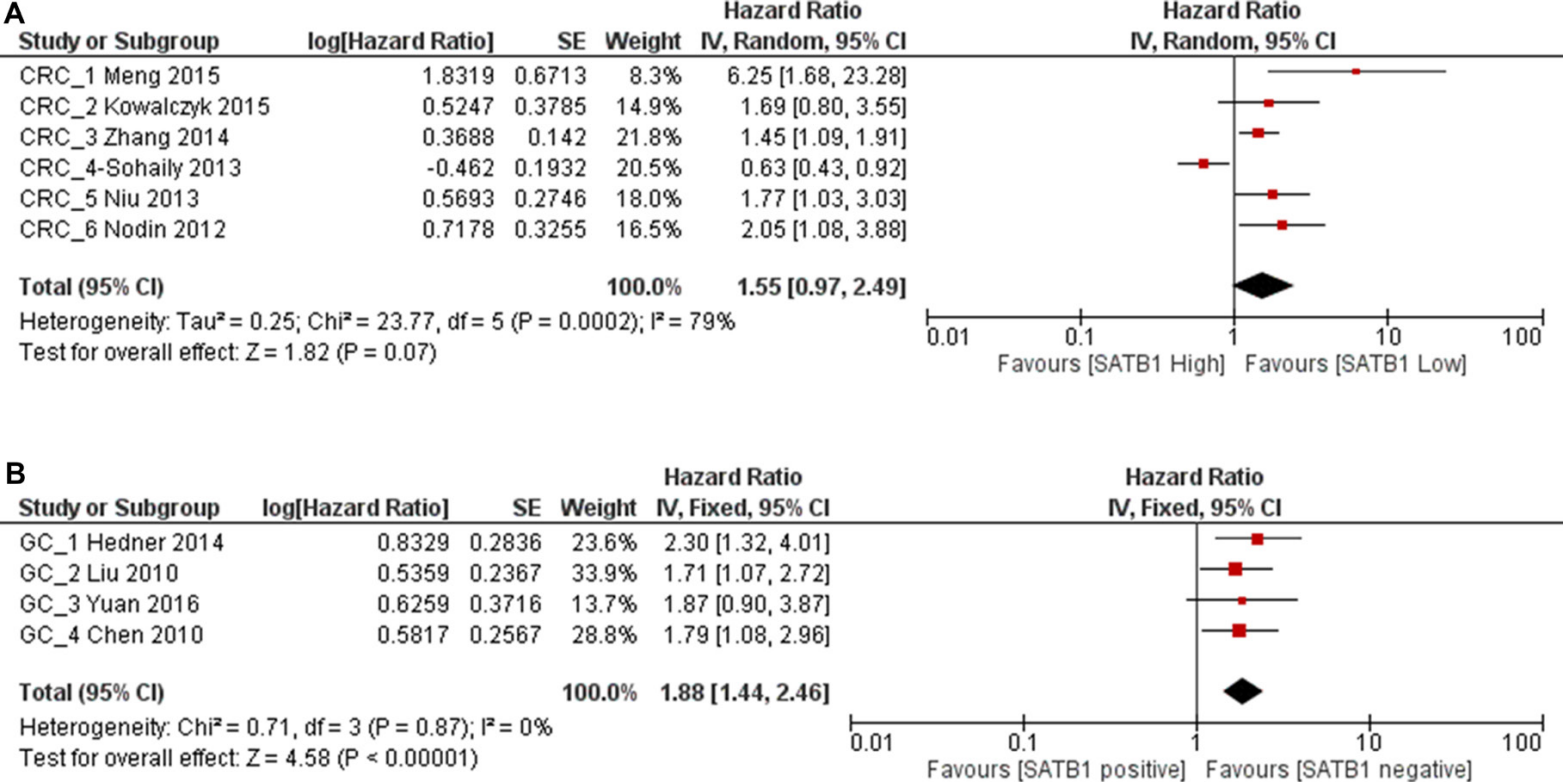
Forest plot of HR for OS associated with SATB1 expression in CRC patients (**A**) and GC patients (**B**).

We analyzed SATB1 expression in CRC and GC patients separately. From 6 studies of CRC patients, although there was a trend of increased mortality for SATB1 overexpression in CRC patients with combined HR 1.55, it was not statistically significant (1863 patients, 95% CI 0.97–2.49, *p* = 0.07) (Figure 6A). In GC patients, the combined HR from 4 studies was 1.88 (455 patients, 95% CI 1.44–2.46, *p* < 0.00001), which indicated that the overexpression of SATB1 increase mortality by 1.88 fold (Figure 6B). No publication bias was detected by Begg's test (*p* = 0.26) and Egger's test (*p* = 0.29) (Figure 5B). Since there was only one study each regarding esophageal cancer and pancreatic cancer, we did not analyze them individually.

### Correlation between SATB1 expression and clinicopathological characteristics

**Figure 7:**
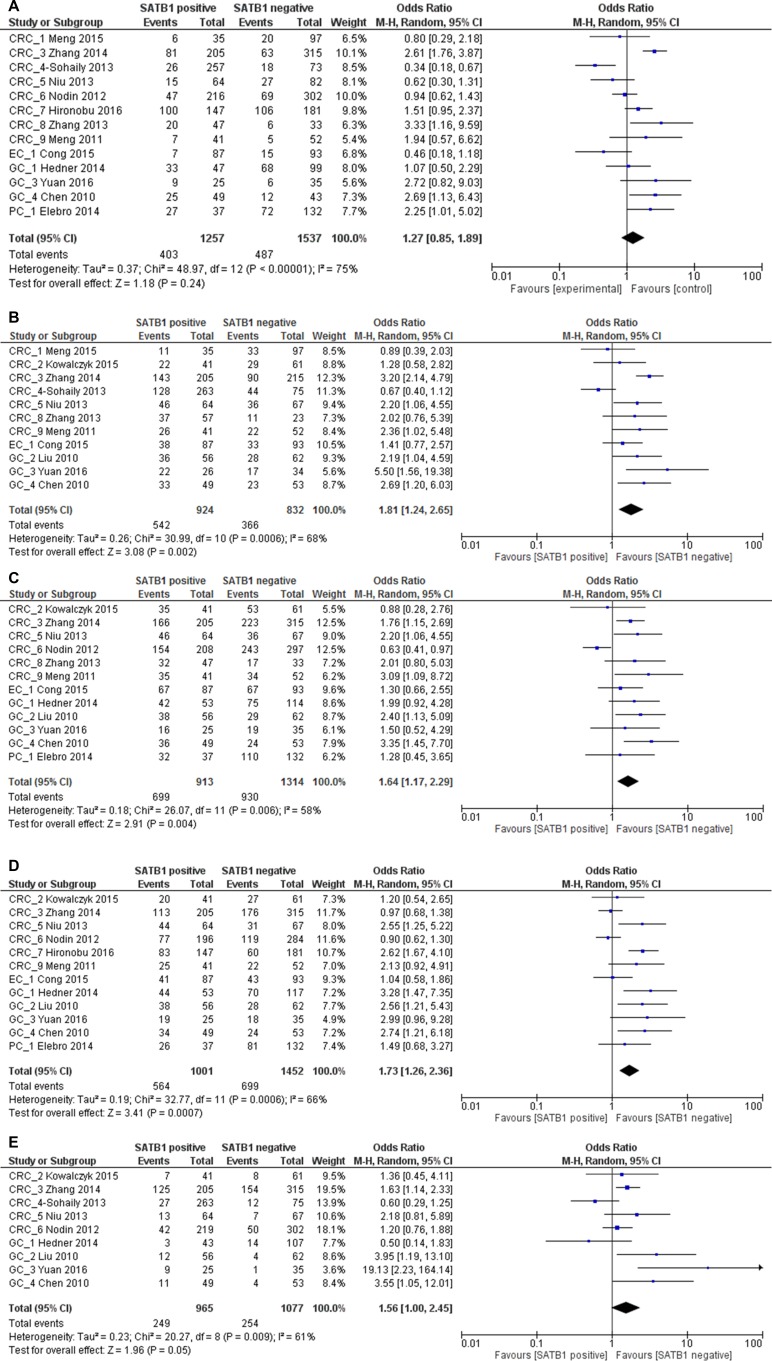
Relation between SATB1 expression and poor differentiation grade (**A**), late TNM stage (**B**), late T3 or T4 stage (**C**), lymph node metastasis (**D**) and distant metastasis (**E**) in gastrointestinal patients.

We showed that SATB1 overexpression increases mortality and recurrence risk in gastrointestinal cancer patients. To further explore the potential cause, we examined the correlation between SATB1 expression and clinicopathological characteristics according to the available data. In all gastrointestinal cancer, there was no clear correlation between SATB1 expression and differentiation grade (2794 patients; pooled OR: 1.27, 95% CI 0.85–1.89, *p* = 0.24) (Figure 7A). However, SATB1 expression was significantly associated with advanced (stage III/IV) TNM stage (1756 patients; pooled OR: 1.81, 95% CI 1.24–2.65, *p* = 0.002), advanced (T3/T4) T stage (2227 patients; pooled OR: 1.64, 95% CI 1.17–2.29, *p* = 0.004), lymph node metastasis (2453 patients; pooled OR: 1.73, 95% CI 1.26–2.36, *p* = 0.0007) and distant metastasis (2042 patients; pooled OR: 1.56, 95% CI 1.00–2.45, *p* = 0.05) (Figure 7B–7E).

**Figure 8:**
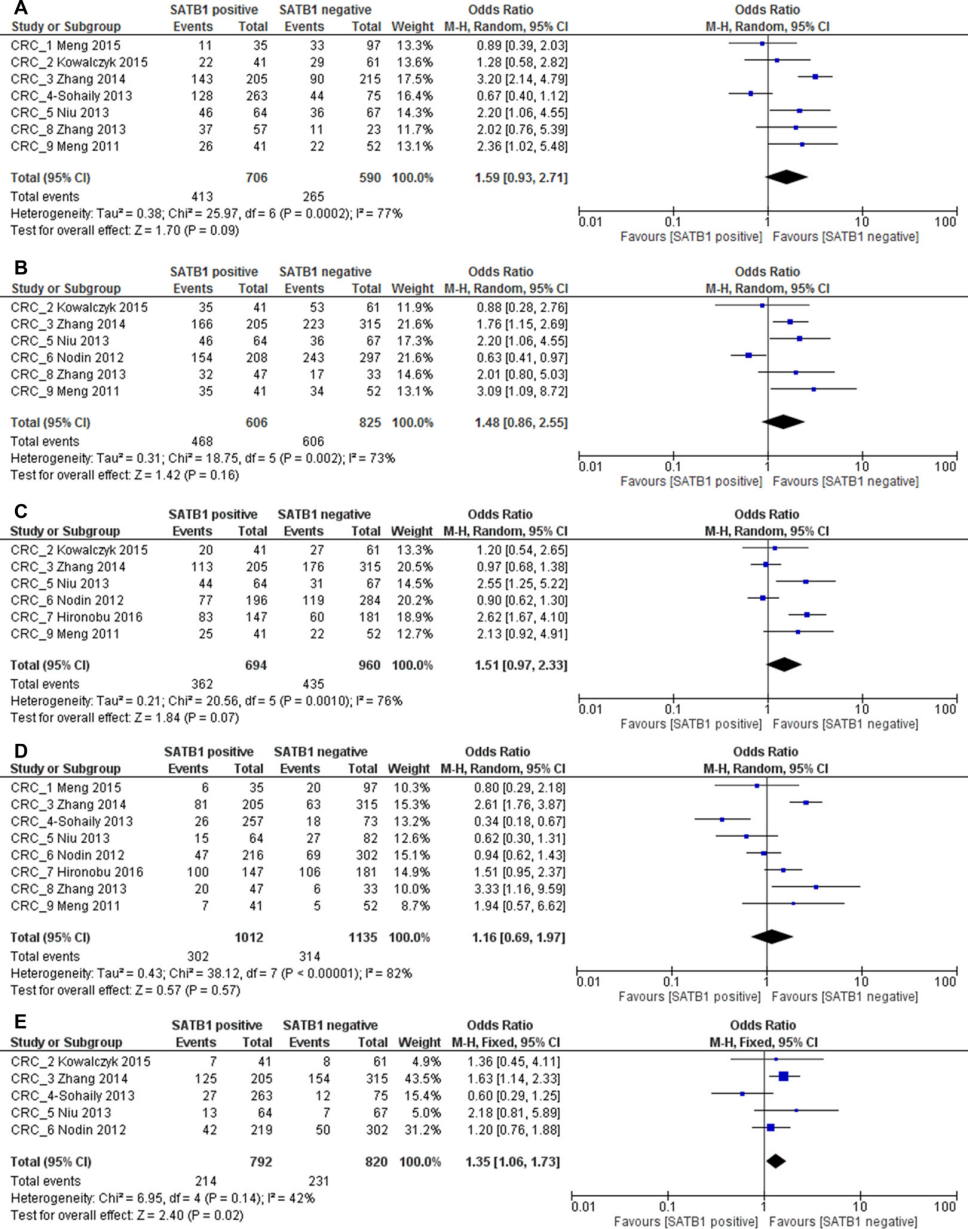
Relation between SATB1 expression and TNM stage (**A**), T stage (**B**), lymph node metastasis (**C**), differentiation (**D**) and distant metastasis (**E**) in CRC patients.

When stratified by cancer type, we found that in CRC no significant correlation was found between SATB1 expression and TNM stage (1296 patients; pooled OR: 1.59, 95% CI 0.93–2.71, *p* = 0.09), T stage (1431 patients; pooled OR: 1.48, 95% CI 0.86–2.55, *p* = 0.16), lymph node metastasis (1654 patients; pooled OR: 1.51, 95% CI 0.97–2.33, *p* = 0.07) and tumor differentiation (2147 patients; pooled OR: 1.16, 95% CI 0.69–1.97, *p* = 0.57). There was a significant correlation between SATB1 expression and distant metastasis in CRC patients, with a pooled OR of 1.35 (1612 patients, 95% CI 1.06–1.73, *p* = 0.02) (Figure 8A–8E).

**Figure 9:**
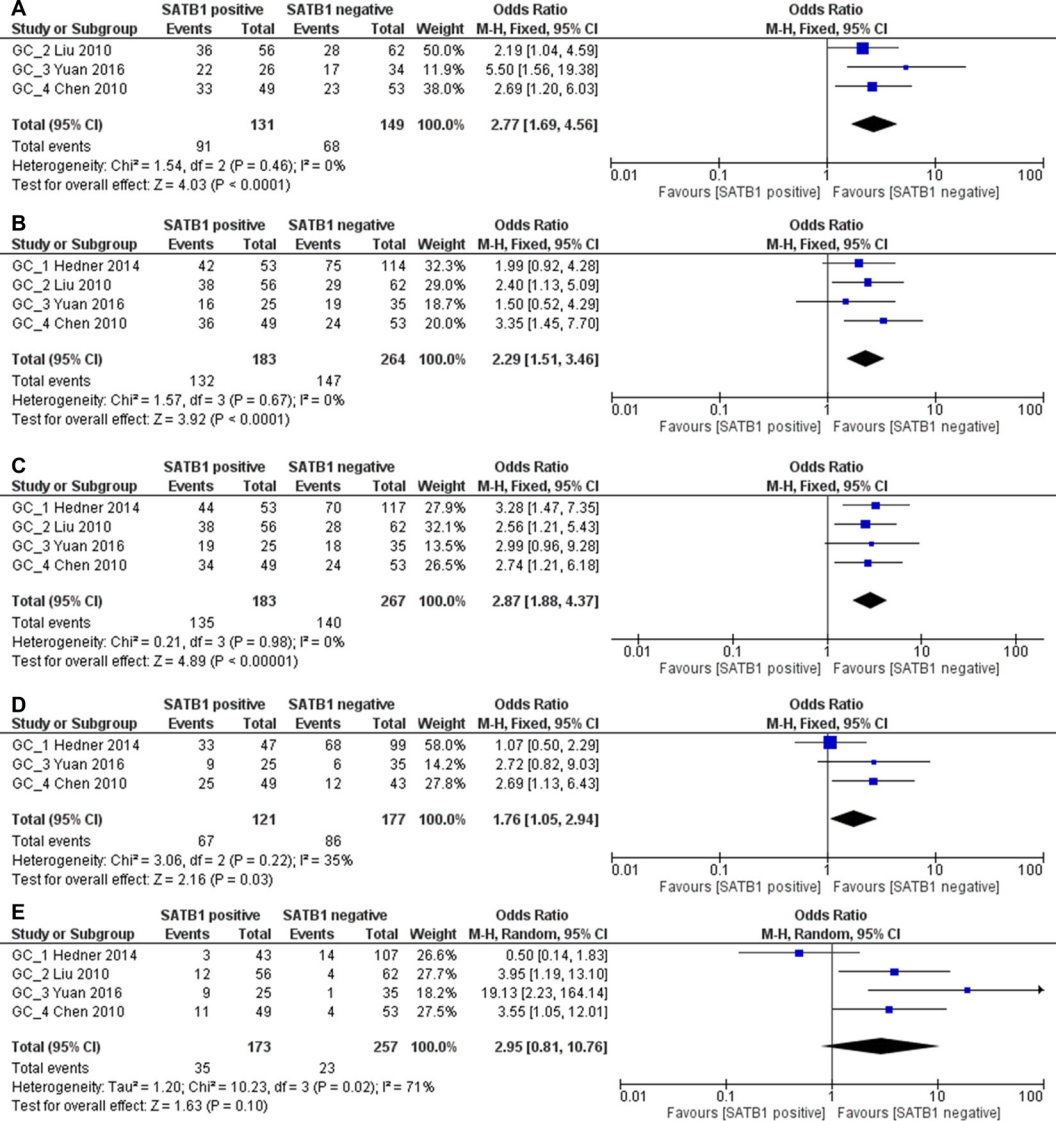
Relation between SATB1 expression and TNM stage (**A**), T stage (**B**), lymph node metastasis (**C**), differentiation (**D**) and distant metastasis (**E**) in GC patients.

However, in GC patients, SATB1 was significantly correlated with advanced (stage III/IV) TNM stage (280 patients; pooled OR: 2.77, 95% CI 1.69–4.56, *p* < 0.0001), advanced(T3/T4) T stage (447 patients; pooled OR: 2.29, 95% CI 1.51–3.46, *p* < 0.0001), lymph node metastasis (450 patients; pooled OR: 2.87, 95% CI 1.88–4.37, *p* < 0.0001) and poor tumor differentiation (298 patients; pooled OR: 1.76, 95% CI 1.05–2.94, *p* = 0.03). No clear correlation was found between SATB1 expression and distant metastasis (430 patients; pooled OR: 2.95, 95% CI 0.81–10.76, *p* = 0.10) (Figure 9A–9E).

### Assessment of publication bias

As shown in Figure 3 and Figure 5, no significant publication bias was detected in all meta-analysis. Therefore, trim and fill method in Stata was not used in this article.

## DISCUSSION

Gastrointestinal cancer is a major global health care problem. Clinical parameters such as TNM stage and lymph node involvement are generally considered as prognostic factors but are insufficient to provide useful information in terms of treatment options. Target therapy is one of the comprehensive treatments in cancer patients. As the discovery of novel targets and the development of new anti-cancer medicines, more patients can benefit from these target therapies. Still, the intrinsic heterogeneity in cancer requires an urgent need to explore and identify new targets in personalized treatment of solid tumors and other types of cancer [31]. The idea that SATB1 is a potential prognosis factor in gastrointestinal cancer patients stemmed from the finding that SATB1 plays a crucial role in the development of colorectal cancer [32], gastric cancer [33] and other types of cancer/solid tumors [34–35]. Although SATB1 overexpression was reported in gastrointestinal cancer and correlated with poor prognosis, the impact of SATB1 expression on overall survival is still inconclusive. In this study, we included 15 studies with 3174 patients and analyzed whether SATB1was a significant prognostic risk factor in gastrointestinal cancer. Based on our evaluation, SATB1 plays an important role in predicting poor prognosis in gastrointestinal cancer patients.

### All gastrointestinal cancer, CRC and GC

In general, SATB1 is overexpressed in cancerous tissue compare to normal adjacent tissue (Supplementary Table 1). First we analyzed the overall influence of SATB1 expression on OS in all gastrointestinal cancer, including 6 studies of colorectal cancer, 4 studies of gastric cancer, one study of esophageal cancer and one study of pancreatic cancer. Our result demonstrated that SATB1overexpression was correlated with a 1.79 (1.34–2.41) fold increase in mortality in all gastrointestinal cancer patients. When stratified by CRC or GC, we found that SATB1 expression (overexpression?) was only associated with a trend of increased mortality in CRC patients, with combined HR 1.55 (0.97–2.49). It is still controversial how the expression of SATB1 can be associated with prognosis of CRC. Within the individual studies included in our meta-analysis, all studies but one [19] showed that SATB1 overexpression is correlated with poor prognosis in CRC patients. In contrast, Al-Sohaily and colleagues [19] found that SATB1 overexpression was correlated with better survival in CRC patients. The reason for this discrepancy might be but not limited to the followings: 1. Biological role of SATB1: SATB1 is a genomic organizer which activates certain gene expression while inhibits others. It may play dual or opposite roles in different circumstances. 2. Heterogeneity between studies included: Sohaily found that the association between loss of SATB1expression and poor OS was even stronger in right colon cancer patients, which implied that although colorectal cancer is usually considered as a single disease, differences still exist between right-sided, left-sided, sigmoid and rectal cancer both in clinical and biological aspects, however in Sun et al [16] and Meng et al [24], they only included rectal cancer patients. Differences in proportion of CRC location might also contribute to the discrepancies; 3. Relatively small sample size: We only found 6 studies that are eligible for evaluating the impact of SATB1 over-expression on OS of CRC patients; more studies may increase the power of this meta-analysis. In GC patients, although we have only 4 studies in this meta-analysis, SATB1 overexpression showed a significant correlation with mortality with a remarkably increased HR (combined HR: 1.88, 95% CI 1.44–2.46, *p* < 0.00001). The result seems consistent and no heterogeneity (*p* = 0.87) or publication bias (*p* = 0.29) was detected.

### Asian and western populations

The ethnic difference between Asian and Western population has been identified and investigated for several years [36]. The difference in clinical characteristics between Asian and Non-Asian population gastric cancer patients was also been well demonstrated by several studies [37–38]. Chen and colleagues [39] retrospectively collected data from a single center in Australia and found that despite similar clinicopathological characteristics and treatment options, Asian gastric cancer patients had a superior survival time than Non-Asian counterparts. It is not clear whether potential ethnic differences in clinical characteristics or tumor biology account for the above results. Surprisingly in our subgroup studies, we found that SATB1 expression was positively correlated with poor OS in Asian population (combined HR: 2.00, *p* < 0.00001). In Western population, this correlation is not statistically significant (combined HR: 1.51, *p* = 0.16). Our result suggested that population-based specificity in genotype and phenotype should be taken into consideration when developing novel target therapies/drug targets.

### Clinical parameters

Clinical parameters such as advanced TNM stage, positive lymph node metastasis, and positive distant metastasis are important factors for poor prognosis in gastrointestinal cancer. *In vitro* study has shown that SATB1 can up-regulate genes that are known to promote cancer cell metastasis to the lung [40]. Clinical study also demonstrated that SATB1 over-expression was correlated with several parameters [41]. In our meta-analysis, we evaluated the relation between SATB1 expression and clinicopathological characteristics. In all gastrointestinal cancer patients, TNM stage (OR: 1.81, 1.24–2.65), T stage (OR: 1.64, 1.17–2.29), lymph node metastasis (OR: 1.73, 1.26–2.36) and distant metastasis (OR: 1.56, 1.00–2.45) are correlated with SATB1 over-expression. Moreover, after stratified with CRC or GC, we found that in GC patients this correlation coefficient is higher (TNM stage OR: 2.77, 1.69–4.56; T stage OR: 2.29, 1.51–3.46; lymph node metastasis OR: 2.87, 1.88–4.37) expect for distant metastasis (OR: 2.95, 0.81–10.76). In addition, SATB1 expression is also significantly correlated with poor tumor differentiation (OR: 1.76, 1.05–2.94). All these evidences suggested that SATB1 could be used to predict prognosis in gastric cancer patients.

### Quality assessment and limitations

According to Table 2, all included studies have high quality scores except one [27] (Supplementary Table 2). There are several limitations in this meta-analysis. First, it is inconclusive whether SATB1 expression correlates with OS in colorectal cancer. Studies have shown that SATB2-homology of SATB1- might serve as a prognostic factor in CRC [42]. Opposite to SATB1, SATB2 expression correlated with better prognosis in CRC patients. Recently Mansour et al has shown that SATB1 and SATB2 play opposing roles in c-MYC expression and progression of colorectal cancer *in vitro* [43]. Therefore, SATB1 and SATB2 expression might be detected together to provide a more accurate evaluation system in prognosis of CRC patients. Second, relatively small number of studies included (15 studies) may lead to a less powerful result in this meta-analysis. Third, although most of the included studies except one [27] used IHC or TMA for IHC to detect SATB1 expression, differences in reagent, staining protocols and cut-off point still exist (Supplementary Table 1). This may cause discrepancies and affect the overall results. High quality score of included studies may mitigate the variations between studies to some extent. Finally, subgroup analysis of association between SATB1 and OS in gastric cancer only included 4 studies, three of which were carried out in Asian population. It might be insufficient to draw conclusions that can be applied to all ethnic groups. Thus, more high quality studies are needed to draw more reliable conclusions.

**Table 2:** Items for quality assessment

Introduction	
1.	Give rationale for study hypothesis and objectives
Materials and Methods	
Patients	
2.	Describe patient characteristics: List all candidate variables (e.g. age, menopause status, disease type, etc)
3.	Describe treatment received by the patients
Specimen	
4.	Describe type of the specimen and control samples
Assay methods	
5.	Describe in details the methods used to detect SATB1 (eg. Quantitative PCR or immunohistochemistry staining, etc).
6.	Manufacturer and catalog number for reagents
7.	Evaluation methods: cut off point determination
8.	Negative and positive control and blind methods applied
Study design	
9.	Give rationale for sample size
10.	Case selection criteria: state inclusion and/or exclusion criteria; whether prospective or retrospective; whether stratification or matching was employed; the period from which cases were taken
11.	Follow-up description: follow-up period or median follow up time
12.	Outcome description: define all clinical endpoints examined
Statistical analysis	
13.	Specify all statistical methods and information (methods to analyze correlation of SATB1 expression and clinical parameters, methods to analyze overall survival and/or disease free survival, *p* value, statistical software applied)
Results	
Data and analysis	
14.	Describe SATB1 expression in gastrointestinal cancer patients and its correlation to standard prognostic variables
15.	Present univariate analyses showing the relation between SATB1 and outcome, with estimated effect (eg. Hazard ratio).
16.	For multivariate analyses, report estimated effects (eg. Hazard ratio) with confidence interval for SATB1, adjusted for other risk factors
17.	Missing data: Describe the missing number value for SATB1 and how to deal with it
Discussion	
18.	Interpret the results in the context of hypotheses and other relevant studies
19.	Discussion of potential confounding factor of the study
20.	Discussion of limitation of the study, clinical value of SATB1 and implication for future investigation

## MATERIALS AND METHODS

### Search strategy

We searched the electronic databases of Cochrane Library, PubMed, Embase and Web of science using the keywords “colorectal cancer/carcinoma”, “colon cancer/carcinoma”, ”rectal cancer/carcinoma”, “gastric cancer/carcinoma”, “stomach cancer/carcinoma”, “esophageal cancer/carcinoma”, “pancreatic cancer/carcinoma” “SATB1”, “special AT-rich sequence binding protein 1” and “prognosis”. The titles and abstracts were screened first to exclude all irrelevant studies. Duplicated studies were then removed and the final inclusion of studies was determined by reading the full text. The citation lists of all eligible articles were screened to further identify other potentially relevant publications.

### Selection criteria

To be eligible for inclusion in this systematic review, a study was required to meet the following inclusion criteria: (1) studies published in English and full text is accessible; (2) studies focused on human primary colorectal, gastric, esophageal or other gastrointestinal cancer; (3) studies provided survival information such as RFS, OS associated with SATB1 expression, otherwise studies were included in clinical parameter analysis; (4) studies provided hazard ration (HR) and 95% CI, or data that could be used to estimate the HRs and 95%CIs, or Kaplan-Meier survival curves with sufficient data to extract HRs and 95%CIs; (5) studies provided correlation of SATB1 overexpression with clinical parameters such as TNM stage, histological grade, lymph node involement, etc.; (6) peer-reviewed and published original articles. Exclusion criteria: (1) no data on survival, or unable to calculate hazard ratios based on data provided, or no data on clinical parameters; (2) letters, comments, reviews or book chapters. When similar studies were published from the same institution, confirm the detailed information from the study or contact with the authors that the patients’ data are not overlapped, otherwise only the most recently published was included to avoid overlap.

### Data extraction and quality assessment

Two reviewers (SZ and XSX) searched and assessed the studies independently. The included studies were chosen by consensus. The following data were recorded from each study: author, year, country, patient number, SATB1 detection method, cut-off value, clinicopathological features, follow-up period, statistical method, hazard ratio of OS and/or DFS. Quality assessment of the included studies was performed by independent reviewers (HL and YXT) according to REMARK (REporting recommendations for tumor MARKer prognostic studies) guideline [44]. We extracted 20 items (Table 1) including information in study design, assay method, outcomes and statistical analysis method to assess the quality of the eligible studies. Each item was scored as follows: 2 points if it is clearly indicated; 1 point if the description is partial or unclear and 0 point if it was not mentioned in the study. The final quality score is presented as a percentage as the sum points of the total 20 items divided by 40. Higher percentages represent better quality of the original study.

### Statistical analysis

HRs and 95%CIs were combined from individual studies to determine the overall effective value of SATB1. Specific calculation methods were applied when HRs and 95% CIs were not provided according to literature [45]. An observed HR > 1 implied SATB1 overexpression was a risk factor for overall survival. Statistical analysis was performed by Cochrane RevMan 5.3.0 (The Cochrane Collaboration, Copenhagen). The χ^2^-square test was used to evaluate heterogeneity between studies. *P*-value < 0.05 is considered significant. If the test of heterogeneity is significant, a combined HR was calculated by random-effects model; otherwise the fixed-effects model was used. The total variation among studies was estimated by I^2^. Publication bias was assessed by funnel plot. Begg's regression and Egger's linear regression [46, 47] method performed by StataSE 12.0 (Stata Corp LP, College Station, Texas, USA) were also used to assess publication bias. *P* > 0.05 suggested that there was no significant publication bias. If *P* < 0.05, “trim and fill” method was used to test the potential influence of unpublished studies on the summary result.

## CONCLUSIONS

This meta-analysis for the first time evaluated the prognostic value of SATB1 expression in gastrointestinal cancer patients. We demonstrated that the expression of SATB1 is a validated prognostic factor for unfavorable outcome in gastrointestinal cancer. SATB1 expression is correlated with classical clinical parameter such as TNM stage, lymph node metastasis, distant metastasis, factors that are associated with a shorter overall survival. The novelty of this study is that it provided insight and evidence for further research to explore SATB1 as a target in cancer diagnosis and personalized treatment.

## SUPPLEMENTARY MATERIALS TABLES


