# Supplementary material for: Prognostic significance of SATB1 in gastrointestinal cancer: a meta-analysis and literature review
Source: Oncotarget. 2017 Apr 5;8(29):48410–23. doi: 10.18632/oncotarget.16867 (PMC5564658; doi:10.18632/oncotarget.16867)
Supplement: Supplementary file 1 [file oncotarget-08-48410-s001.pdf]

Prognostic significance of SATB1 in gastrointestinal cancer: a meta-analysis and literature review

Supplementary Materials

Supplementary Table 1: SATB1 expression rate, dectetion methods and therapeutic informations of studies included in meta-analysis

Study	Journal	Cancer Type	Treatment	SATB1 expression (cancer vs normal)	Detection method and reagent
**Sun** [16]	Cancer biology & Therapy	Rectal Cancer	Curative operation +/- preoperative radiotherapy	38% vs 21%	IHC, monoclonal anti-SATB1 (1:250; Epitomics), 4° overnight
**Kowalczyk** [17]	Tumor Biology	CRC	Curative operation	Nuclear: 84.3% vs 71.8%<br>Cytoplasmic: 30.4% vs 7.7%	IHC, anti−SATB1 (1:100, EPR3951, GeneTex), room temperature 20 min
**Zhang** [18]	Plos One	CRC	Curative operation	43% vs 1.8%	IHC, anti−SATB1 (1:100, PRS4631, Sigma−Aldrich)
**Al-Sohaily** [19]	Histopathology	CRC	Curative operation	Nuclear: 78% vs 98.5%<br>Cytoplasmic: 18.7% vs 2%	IHC, anti−SATB1 (1:75, catalogue 611182; BD Biosciences), room temperature 60 min
**Niu** [20]	APMIS	CRC	Curative operation	48.9% vs 16.8%	IHC, polyclonal anti−SATB1 (1:200; Abcam), 4° overnight
**Nodin** [21]	Diagnostic Pathology	CRC	Curative operation	42% vs 12.5%	IHC, monoclonal anti−SATB1 (1:100, Clone EPR3895, Epitomics)
**Hironobu** [22]	Anticancer Research	CRC	Curative operation	N/A	IHC, monoclonal anti−SATB1 (1:100, ab92307; Abcam),4° overnight
**Zhang** [23]	Plos One	CRC	Curative operation	58.8% vs 2.5%	IHC, anti−SATB1 (1:100, BD Biosciences), 4° overnight
**Meng** [24]	International Journal of Colorectal Dis	Rectal Cancer	Curative operation	44.1% vs 25.8%	IHC, anti−SATB1 (1:250; Epitomics), 4° overnight
**Hedner** [25]	Virchows Arch	GC & EC	R0 resection:122 (69.7)<br>R1 resection:34 (19.4)<br>R2 resection:19 (10.9)	31.2% in tumor<br>40.5% in metastases<br>0% in normal tissue	IHC, monoclonal anti−SATB1 (1:100, Clone<br>EPR3895, Epitomics)
**Lu** [26]	Oncology Report	GC	Surgical resection	N/A	IHC, polyclonal anti−SATB1 (1:200, Abcam), 4° overnight
**Yuan** [27]	European Review for Medical and Pharmacological Sciences	GC	Surgery (100%) and adjuvant chemotherapy (70%)	N/A	QPCR<br>2−ΔΔCT
**Chen** [28]	APMIS	GC	Surgical resection	48% vs 18.6%	IHC, polyclonal anti−SATB1 (1:200, Abcam), 4° overnight
**Cong** [29]	Diseases of the Esophagus	EC	Curative operation	48.3% vs 7.8%	IHC, polyclonal anti−SATB1 (1:200, Abcam), room temperature 2h
**Elebro** [30]	Journal of Translational Medicine	PC	Curative operation +/− adjuvant therapy	21.9% in cancer	IHC, anti−SATB1 (clone EPR3895, Epitomics)

Supplementary Table 2: Details of quality score for included studies

Study	Item 1	Item 2	Item 3	Item 4	Item 5	Item 6	Item 7	Item 8	Item 9	Item 10	Item 11	Item 12	Item 13	Item 14	Item 15	Item 16	Item 17	Item 18	Item 19	Item 20	Sum	Quality Score%
CRC_1	2	1	2	2	2	1	2	2	0	1	2	2	2	2	2	2	1	2	1	1	32	80%
CRC_2	2	2	2	2	2	2	2	2	0	1	1	2	2	2	0	2	1	1	1	2	31	78%
CRC_3	2	2	1	1	2	2	1	2	0	1	0	2	2	2	0	2	1	2	2	2	29	73%
CRC_4	1	2	2	2	2	2	2	2	0	1	1	2	2	2	2	2	1	1	2	1	32	80%
CRC_5	2	2	1	2	2	2	2	2	0	1	2	2	2	2	2	2	1	2	2	2	35	88%
CRC_6	2	2	1	2	2	2	2	2	0	1	2	2	2	2	2	2	1	1	1	1	32	80%
CRC_7	2	2	2	2	2	1	2	2	0	1	2	2	2	2	2	2	1	2	1	1	33	83%
GC_1	1	2	2	2	2	2	2	2	0	1	2	2	2	2	0	2	1	2	2	2	33	83%
GC_2	2	2	2	2	2	2	2	2	0	1	0	2	2	2	2	2	1	1	2	2	33	83%
GC_3	2	2	2	0	2	0	2	0	0	1	0	2	2	2	2	0	1	2	1	1	24	60%
GC_4	2	1	2	2	2	2	2	1	0	1	2	2	2	2	0	2	1	2	2	2	32	80%
EC_1	1	2	2	2	2	2	2	2	0	1	0	2	2	2	2	1	1	1	2	1	30	75%
PC_1	1	2	2	2	2	1	2	2	0	1	0	2	2	2	2	2	1	2	2	1	31	78%
